# Blood Pressure and T-Tau in Spinal Fluid Are Associated With Delayed Recall in Participants With Memory Complaints and Dementia of the Alzheimer’s Type

**DOI:** 10.3389/fnagi.2021.652510

**Published:** 2021-10-28

**Authors:** Knut Asbjorn Hestad, Peter Otto Horndalsveen, Knut Engedal

**Affiliations:** ^1^Department of Health and Nursing Science, Faculty of Social and Health Sciences, Inland Norway University of Applied Sciences, Elverum, Norway; ^2^Department of Research, Innlandet Hospital Trust, Ottestad, Norway; ^3^Department of Old Age Psychiatry, Innlandet Hospital Trust, Ottestad, Norway; ^4^Norwegian National Advisory Unit on Ageing and Health, Vestfold County Hospital Trust, Tønsberg, Norway; ^5^Department of Geriatric Medicine, Oslo University Hospital, Oslo, Norway

**Keywords:** blood pressure, aging, systolic, total-tau, beta-amyloid, antihypertensive medication, cognition

## Abstract

**Objective**: The aim of the study was to determine if systolic blood pressure (SBP), total-tau (t-tau), and beta-amyloid (Aβ) in the cerebral spinal fluid (CSF) were associated with the results on the Consortium to Establish a Registry for Alzheimer’s Disease Word List (CERAD-WL) immediate and delayed recall, and the Mini Mental State Examination (MMSE) in “younger” older adults, controlling for age and sex.

**Method**: We included 72 participants, mean age: 62.9 (SD 8.6, range 41–76) from a Norwegian memory clinic; eight were diagnosed with subjective cognitive decline, 32 with mild cognitive impairment (MCI), 30 with dementia of the Alzheimer’s type (DAT), and two with combined DAT and vascular dementia (VaD). Data were examined in three fitted multiple linear regression models using the CERAD-WL immediate and delayed recall, and MMSE as dependent variables; and SBP, t-tau, and Aβ as independent variables, controlling for age and sex.

**Results**: The strongest associations were found in the model using CERAD-WL delayed recall as the dependent variable, where 45% of the variance was explained (standardized Beta = −0.313, *p* = 0.004 for t-tau and standardized Beta −0.238, *p* = 0.01 for SBP). The unique contribution of age was close to 8%, t-tau close to 7%, and SBP above 5%. When cardiovascular medication was entered into the analysis, the explained variance increased to 51% and Aβ became significant (standardized Beta = 0.216, *p* = 0.03). Participants on this medication exhibited worse performance on CERAD-WL delayed recall than those who were not on medication. Age (7%), t-tau (6%), and SBP (5%) showed the same unique contribution, whereas medication contributed 6% and Aβ contributed 4%. CERAD-WL immediate recall, and MMSE yielded similar findings, but explained variance was poorer for these two variables.

**Conclusions**: Both elevated SBP and t-tau were associated with poorer cognitive performance, especially delayed recall. Those on cardiovascular medication were more impaired than were participants who were not on this medication—a finding that probably reflected cerebral incidents in the medicated group.

## Introduction

People with high blood pressure (BP), especially high systolic BP (SBP) in their middle age, have a higher risk of developing dementia in old age than do people with normal BP (Gottesman et al., 2014). This is the case for the development of both dementia due to Alzheimer’s disease (DAT) and Vascular dementia (VaD; Launer et al., 1995; Kilander et al., 1998; Swan et al., 1998a,b; Yamada et al., 2003; Whitmer et al., 2005; Kimm et al., 2011; Livingston et al., 2017). Some studies have found that low BP rather than high BP is associated with poor cognitive performance and dementia among people above 80 years of age (Hestad et al., 2005). Blood pressure is primarily linked to vascular damage and is the most important risk factor for stroke. Many studies have shown, however, that vascular dysfunction contributes to both VaD and DAT (Zlokovic, 2011; Snyder et al., 2015). Nelson et al. (2012) has proposed a “two-hit vascular” model wherein the vascular damage first creates an initial injury, which can create an accumulation of toxins related to Alzheimer’s disease in the brain. In cognitively intact older individuals (mean age 72), an interaction has been found between cerebral blood flow in brain areas that are affected in Alzheimer’s disease and beta-amyloid peptide (Aβ) accumulation. In a linear regression model, this interaction predicted memory performance in the older subjects, controlling for age, education, and sex (Bangen et al., 2017). Another study of participants without dementia (age 55–91) found that individuals with a phospho-tau (p-tau) positive profile exhibited elevated pulse pressure relative to those with negative profiles. Systolic contributions were seen only in the very old participants (80 years or older), whereas in the younger participant (55–79 years of age), it was a diastolic contribution (Nation et al., 2015).

Different types of biomarkers have been linked to the development of dementia, especially to DAT. It is well known and accepted that the deposition of Aβ and an increase in circulating total-tau (t-tau) t-tau and p-tau in the cerebral spinal fluid (CSF) are hallmarks of DAT (Fagan et al., 2006; Nelson et al., 2012). Nelson et al. conclude that even if Aβ plays a key role in the pathology of DAT, cognitive impairment has a stronger association with neurofibrillary tangles in the brain (p-tau). t-tau may be a marker of neuronal injury and may increase in non-AD pathology. The brain’s concentration of Aβ shows an inverse correlation with Aβ, measured in CSF in DAT (Fagan et al., 2006), which has been explained by the brain’s retention of the Aβ (Blennow et al., 2010). There is an increase of both t-tau and p-tau in the brain in DAT. It is suggested that an increase in t-tau reflects an increase in mild cognitive impairment (MCI) progressing to DAT, whereas p-tau may indicate a phosphorylation state of tau and formation of neurofibrillary tangles in the brain (Blennow et al., 2010). However, an increase of both t-pau and p-tau examined in CSF is found in forms of dementia other than DAT and in healthy older adults (Blennow et al., 1995; Sjögren et al., 2001).

We wanted to follow up on this association between vascular factors and dementia to examine whether SBP could be associated with cognitive performance together with and in addition to Aβ, t-tau, and p-tau, as measured in CSF. We wanted to examine these associations in relation to immediate and delayed recall on a word list as an indicator of memory decline—often the first symptom of dementia and the MCI diagnosis. Our hypothesis is that BP together with tau pathology will be associated with cognitive performance, as tau pathology is associated with the conversion of MCI to dementia.

Research aim: To determine the associations of BP, t-tau, p-tau, and Aβ in CSF with immediate and delayed recall of a word list.

## Materials and Methods

### Participants

Patients were recruited from the Memory Clinic of Innlandet Hospital Trust. As part of the standardized and comprehensive clinical evaluation, patients were examined with neuropsychological tests at the clinic, and CSF was drawn at the Department of Neurology.

The Memory Clinic assesses people with memory problems and dementia—patients referred from general practitioners in the catchment area of the hospital. Of the 996 patients registered in the clinic’s database, 964 had been examined with the Consortium to Establish a Registry for Alzheimer’s Disease Word List (CERAD-WL; Morris et al., 1988; Hankee et al., 2016) and 880 with the Mini mental status examination (MMSE; Folstein et al., 1975). BP had been measured in 737 patients, p- and t-tau in 102 patients, and Aβ in 100. In total, 78 patients had complete data for BP, Aβ, p- and t-tau, and memory testing with CERAD-WL. One of the 78 lacked a score on the MMSE. Six participants with either a diagnosis of pure VaD or Parkinson’s disease were excluded from the analyses. The raw scores from the tests were used in the analyses.

### Diagnosis

The dementia diagnoses were based on the International Classification of Diseases (ICD-10) criteria for research [World Health Organization (WHO), 1993] and made by experienced geriatric psychiatrists, usually in consensus meetings. The Winblad criteria were used for MCI (Winblad et al., 2004). Patients who complained about cognitive decline but showed no cognitive impairment on neuropsychological tests were given the diagnosis of Subjective cognitive decline (SCD), in accordance with the Jessen criteria (Jessen et al., 2014).

### Lumbar Puncture

Lumbar puncture with the examination of Aβ, t- and p-tau was conducted according to the Norwegian national guidelines (Braekhus et al., 2011; Knapskog et al., 2017).

The lumbar punctures were carried out between 9 a.m. and 11 a.m. Spinal fluid was collected in cryotubes and was centrifuged for 10 min at 2,000 *g* within 30 min of collection. Samples were either sent to the laboratory on the same day or frozen at −20 degrees Celsius and later sent in a frozen state. They were analyzed using the Innotest kit (Innogenetics, Ghent, Belgium) for the three biomarkers (See Table 1 for absolute values and number of patients with values outside the reference values of the laboratory: Aβ42, t-tau, and p-tau). We also analyzed p-tau, but this value was not used in the regression due to its high correlation with t-tau. For more details regarding the biomarkers in CSF, see Knapskog et al. (2017).

**Table 1:** Overview of age, sex, systolic and diastolic blood pressure, beta-amyloid, total and phosphorylated tau (t-tau and p-tau), the Consortium to establish a registry for Alzheimer’s Disease word list task (CERAD-WL) immediate and delayed recall, and the Mini Mental State Examination (MMSE).

Diagnosis	N	Age (SD)	Sex Women/men	Systolic Blood Pressure in MMHG (SD)	Diastolic Blood Pressure in MMHG (SD)	Beta-amyloid Mean (SD) [normal/ pathological]	total-tau Mean (SD) [normal/ pathological]	phospho-tau Mean (SD) [normal/ pathological]	CERAD-WL, immediate recall, Mean raw score (SD)	CERAD-WL, delayed recall, Mean raw score (SD)	MMSE, Mean raw score (SD)
Subjective cognitive decline	8	55.5 (10.4)	4/4	134.4 (14.03)	84.5 (7.7)	869.0 (175.6) [87.5/12.5]	372.5 (29.7) [75/25]	58.1 (37.5) [75/25]	18.5 (3.2)	5.5 (1.3)	27.3 (2.0)
Mild Cognitive impairment	32	62.4 (8.9)	13/19	138.1 (17.9)	85.3 (8.8)	833.3 (295.0) [78.1/21.9]	334.3 (177.7) [81.3/18.8]	53.1 (22.5) [90.4/9.6]	16.0 (4.2)	3.8 (2.2)	26.1 (3.1)*
Dementia of Alzheimer’s type	30	64.9 (6.8)	16/14	145.1 (18.5)	86.7 (10.4)	555.4 (133.1) [43.3/56.7]	662.5 (233.2) [16.7/83.3]	99.4 (42.2) [26.7/73.3]	13.3 (4.1)	2.1 (2.0)	24.1 (3.3)
Combined dementia of Alzheimer’s and Vascular type	2	69.5 (0.7)	0/2	154.0 (7.1)	94.0 (12.7)	571.5 (12.0)	472.5 (31.8)	65.5 (7.8)	12.5 (0.7)	1.0 (0.0)	20.5 (2.1)

*One subject is missing. Percentage of participants with [normal/ pathological] values. See Knapskog et al. (2017) for more details regarding Aβ, t, and p-tau.

### The Final Sample

Seventy-two participants had been measured on Aβ, t-tau, p-tau, BP, and the 10-word CERAD-WL, both immediate and delayed recall. In addition, we included the MMSE. The sample consisted of eight patients with SCD, 32 with MCI, 30 with DAT, and two with a combination of DAT and VaD. People with other types of dementia were excluded from the study (See Table 1 for demographics and mean values).

BP had been measured by a study nurse in a clinical setting after the seated patients had become comfortable with the situation—about 30 min into the consultation. If BP were evaluated as high, if the patient seemed to be stressed, or if there appeared to be a white-coat syndrome operating, BP was measured again. The last measurement was then entered into the patient’s record.

### Statistical Analyses

We fitted three multiple linear regression models using SPSS (25) with the cognitive tests (the immediate and delayed recall of the CERAD-WL, and the MMSE) as dependent variables, and t-tau, Aβ, SBP, age, and sex as independent variables in the different analyses. It was checked for deviance from the normal distribution, inspecting PP plots and scatterplots of the dependent variables.

## Results

The highest BP, highest values of p- and t-tau, and lowest values of Aβ were seen in the dementia group(s) (See Table 1).

Because t-tau and p-tau showed a correlation of 0.8, we decided to leave p-tau out of the regression. And because SBP and diastolic BP (DBP) showed a correlation of 0.7, we decided to remove DBP from the regression.

In the regression model using CERAD-WL delayed recall as the dependent variable, all the independent variables except sex showed a higher correlation than 0.3 with the dependent variable. None of the correlations with the dependent variable were above 0.6. We checked for multicollinearity, examining tolerance and variable inflation factor. No values of tolerance were less than 0.10 and no values of variable inflation factor were above 0.10. No outliers were found in our examination of the data.

For CERAD-WL delayed recall, 45% (Adjusted R^2^) of the variance was explained, and the model showed a highly significant result (ANOVA; *F* = 12.7; *p* < 0.0001). The standardized Beta coefficient for age was 0.304 (*p* = 0.002), 0.238 (*p* = 0.01) for SBP, and 0.313 (*p* = 0.004) for t-tau. Sex and Aβ had no significant influence on the standardized Beta (See Table 2). The results were similar for p-tau in a separate analysis (p-tau *p* = 0.007; Age *p* = 0.001, SBP *p* = 0.037). However, in this analysis, the Aβ also showed a significant result (*p* = 0.047).

**Table 2:** Standardized Beta, t-value, p-value, partial correlation, and the squared semipartial correlation to indicate the unique variance explained by each variable for the independent variables.

	Standardized Coefficients Beta	*t*-value	Significance, *p*-value	Partial correlation	Semipartial correlation squared
**CERAD-word list, delayed recall**					
Total tau	−0.313	−2.985	**0.004**	−0.345	0.069
β-amyloid	0.179	1.814	0.074	0.218	0.025
Systolic blood pressure	−0.238	−2.594	**0.012**	−0.304	0.052
Sex	0.035	−0.383	0.703	0.047	0.001
Age	−0.304	−3.200	**0.002**	−0.366	0.079
**CERAD-word list, immediate recall**			
Total tau	−0.281	−2.241	**0.028**	−0.266	0.055
β-amyloid	0.054	0.457	0.650	0.056	0.002
Systolic blood pressure	−0.241	−2.198	**0.031**	−0.261	0.053
Sex	−0.013	−0.117	0.907	−0.014	0.0001
Age	−0.191	−1.692	0.095	−0.204	0.031
**Mini Mental Status Examination**			
Total tau	−0.325	−2.15	**0.014**	−0.298	0.073
β-amyloid	0.074	0.608	0.546	0.075	0.004
Systolic blood pressure	−0.178	−1.579	0.119	−0.192	0.029
Sex	0.084	0.756	0.452	0.093	0.007
Age	−0.080	−0.683	0.497	−0.084	0.005

*Significant results are shown in bold*.

Examining the unique contribution of the significant values using the squared value of the semipartial correlation (called PART in SPSS) coefficients as an indicator, we found that age contributed close to 8% (7.9%), t-tau close to 7% (6.9%), and SBP more than 5% (5.2%). We also sought to determine if there were any interactions among t-tau, SBP, and age (See Table 2). No interactions were found among the independent variables.

For the CERAD-WL immediate recall, 22% (Adjusted R^2^) of the variance was explained (ANOVA result; *F* = 5.1; *p* = 0.001). The standardized Beta coefficient was −0.241 for SBP (*p* = 0.03) and −0.281 (*p* = 0.03) for t-tau. In determining the unique contribution of the significant values using the squared value of the semipartial correlation coefficients as an indicator, t-tau and SBP were found to contribute above 5% each (See Table 2).

For MMSE, 19% (Adjusted R^2^) of the variance was explained. The model showed a significant ANOVA result; *F* = 4.2; *p* = 0.002. The standardized Beta coefficient was −0.262 and was significant for t-tau (*p* = 0.045). The unique variance explained by t-tau was above 7% (See Table 2).

To examine the possible influence of medication, we included medications related to the cardiovascular system in the Anatomical Therapeutic Chemical (ATC) classification in the linear regression, with all medications involving C1–C10. Thirty-six patients, half of the population studied, were on such medications. There were only small changes. The explained variance for the delayed recall of the CERAD-WL increased to 51% and Aβ became significant (Standardized Beta = 0.216, *p* = 0.03). Participants on medication performed worse on the delayed recall of the CERAD-WL than did those who were not on such medication (Standardized Beta, −0.286, *p* = 0.004). The CERAD-WL delayed recall mean was 3.8 (*SD* = 2.4) in the non-cardiovascular medicated group and 2.6 (*SD* = 2.0) in the medicated group. There were no significant differences in medication use across diagnoses (Pearson Chi-Square, *p* = 0.3). Age (7%), t-tau (6%), and SBP (5%) still contributed to the regression with the same unique percentages; medication contributed with 6% and Aβ with 4%.

For the CERAD-WL immediate recall and the MMSE, no significant influence was seen in relation to cardiovascular medication.

**Figure 1:**
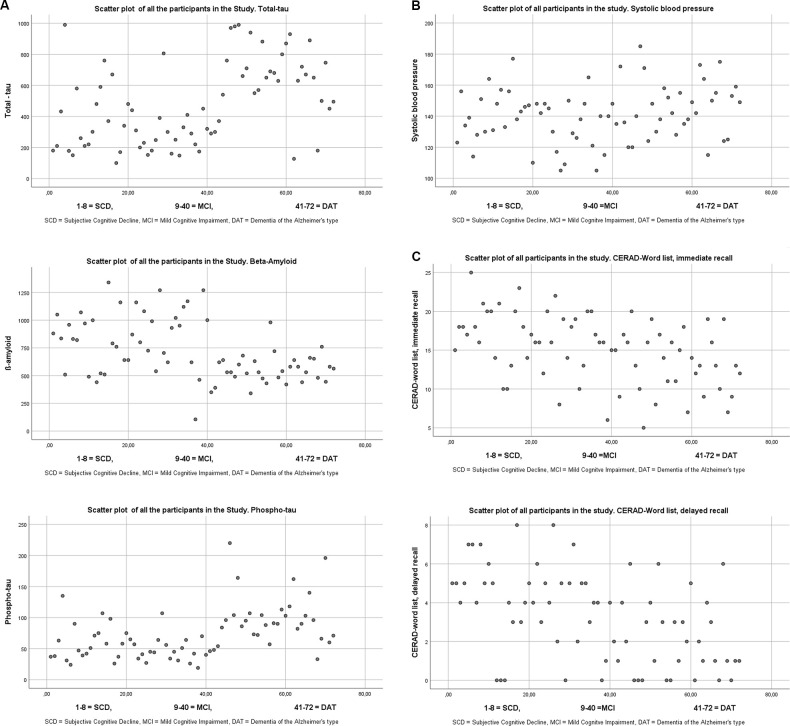
**(A)** Scatterplots of the three different biomarkers from cerebro spinal fluid. The cutoffs for the three biomarkers according to the laboratory (See Knapskog et al., 2017) were for total tau <300 for patients under 50 years, <450 for patients ages 50–69, and <500 for those 70 years and older. It is only total-tau that has age-specified norms. Cut off for β-Amyloid >550, and for phospho tau <80. **(B)** Scatter plot of Systolic blood pressure. **(C)** Scatter plot of the CERAD-Wordlist.

Individual results of the three biomarkers can be seen in Figure 1.

## Discussion

Both SBP and t-tau were significantly associated with cognition, especially for delayed recall of the CERAD-WL. It was the combination of BP and t-tau that showed the strongest association with poor performance on CERAD-WL delay recall, controlling for age and sex. No interaction existed between the two variables, however; neither was there any interaction with age. Our findings do fit with the theory that delayed declarative memory is particularly associated with t-tau and SBP in the aging process. This impairment is associated with elevated SBP and elevated t-tau proteins in CSF. For the CERAD-WL, delayed recall, both SBP, and t-tau made unique contributions (together with age), and the model explained 45% of the variance; when we included cardiovascular medication, 51% of the variation was explained. Patients on antihypertensive medication performed worse on the delayed recall (but not on immediate recall and the MMSE), than those who were not on such medication. We cannot claim that their performance deficits were due to medication, this is probably not the most likely explanation either. High blood pressure is the number 1 reason for cerebrovascular incidents. In this study, we do not have complete data on possible vascular angiopathies, but white matter disorders in the brain are frequent in people with DAT and MCI (Nasrabady et al., 2018; van den Berg et al., 2018; Sweeney et al., 2019). Thus, patients on antihypertensive medication performed worse than did those without medication, most likely because they have a more severe vascular condition. This interpretation of our results is consistent with that of den Heijer et al. (2005), who found that persons treated for hypertension demonstrated more severe atrophy of the brain on MRI, not because of the medication, but because of their initial condition. In a large study that evaluated the effect of intensive BP on the risk of dementia, there was a tendency for reduced risk of dementia and a significant reduced risk of developing MCI (Williamson et al., 2019). In den Heijer et al. (2005) MRI data predicted increased hippocampal atrophy among participants who had exhibited higher DBP 5 years earlier; in persons treated for hypertension, low diastolic pressure was related to cerebral atrophy. In our study, it was high SBP that was associated with cognitive impairment.

That t-tau is associated with cognitive impairment and dementia is not a new finding; many researchers have reported this association (Nelson et al., 2012). Although the highest BP was seen among the dementia patients, they were older than were those with SCD and MCI. The distribution of BP relative to age is in accordance with research we have previously published with the same type of memory-clinic patients (Hestad et al., 2020a,b).

The dementia group in our study was diagnosed with DAT. The largest diagnostic group was MCI. In the SCD and MCI groups, there were a relatively small number of persons with abnormal t-tau values in the CSF compared to those with DAT. SCD is relatively common in the aging process, and patients with that diagnosis often show unspecific symptoms; the majority are unlikely to have a neurodegenerative disease. Among persons with a clinical MCI diagnosis, some will convert to dementia, but some will revert to normal functioning over the next few years (Ritchie et al., 2001). If we had used a biological definition of MCI due to an etiological neurodegenerative disorder rather than the clinical Winblad criteria, we would probably have found fewer patients with MCI but a larger proportion with pathological values of Aβ and tau (Rozzini et al., 2007; Schmidtke and Hermeneit, 2008; Albert et al., 2011; Julayanont et al., 2014; Jack et al., 2018). Some of the patients with SCD and MCI diagnoses who were in our study may have experienced poorer cognition due to normal aging. Given our research question, however, we examined the relationships among BP, Aβ, and tau proteins and their relationship with cognitive functioning independent of diagnosis. It is widely known that high BP is a risk factor for stroke, and it has been noted that people with hypertension are at risk for memory deficits and an increase in t-tau (Glodzik et al., 2014).

In this study, we examined both the shared and unique variance of SBP and t-tau relative to cognitive performance SBP and t-tau correlated positively in our study. The finding was significant, even though the correlation was small to moderate (*r* = 0.24). This finding could fit with the “two-hit vascular model”, which implies that vascular implications may result in Alzheimer’s deposits (Zlokovic, 2011). However, there was no interaction between the two variables. This may suggest these two factors contribute independently to cognitive dysfunction. Thus, on the basis of the statistical models in the study, it may be argued that t-tau and BP contribute independently, not interactively to CERAD-WL scores, and possibly do not per see fit with the two-hit vascular model. The low correlation between these variables may suggest that there are some associations between BP (possible vascular pathology) and t-tau (neuronal degeneration), and the non-significant interaction suggests that they do not work together to produce memory deficits in AD. Vascular lesions are very common in the AD trajectory, with over 80% of cases showing comorbid pathologies. The vascular pathology probably makes the clinical manifestation of neuronal degeneration at an earlier stage than if it had not been there. Most participants in this study had relatively milder forms of cognitive deficits, with a mean MMSE of 25.1. The BP and biomarkers were examined at the time of diagnosis.

The majority of participants in this study were younger older people, and we do not know if the results hold up for very old people with dementia and MCI. Previous studies have found that BP changes with age, and in very old people, low BP has been found to be associated with poorer cognitive performance.

### Limitations

This study included a small sample, and because it was cross-sectional, we could not examine change over time. The patients are referrals to a specialist care unit for evaluation and may not represent a typical population of younger persons with major and mild cognitive impairment.

### Conclusion

Both elevated SBP and t-tau are related to delayed recall of the CERAD-WL. Those on cardiovascular medication were more impaired than were participants who were not on this medication—a finding that probably reflected more initial cerebral incidents in the medicated group. The practical implication may be that better control of BP can stop this development, but it must be done before any cerebral deficits occur.

## Data Availability Statement

The data analyzed in this study was subjected to the following licenses/restrictions: The data was collected from the Norwegian Register of Persons Assessed for Cognitive Symptoms (NorCog). The data can be available after approvement from the board of the database. Requests to access these datasets should be directed to Marit Nåvik, naam@sthf.no.

## Ethics Statement

The studies involving human participants were reviewed and approved by Regional ethics committee for medical and biological research (REK: 2019/316). The patients/participants provided their written informed consent to participate in this study.

## Author Contributions

KH initiated the study, but all authors took part in the planning process. KH did all the statistical analysis and wrote the first draft of the manuscript. All authors took part in the writing process and approved the submitted version of the manuscript.

## Conflict of Interest

The authors declare that the research was conducted in the absence of any commercial or financial relationships that could be construed as a potential conflict of interest.

## Publisher’s Note

All claims expressed in this article are solely those of the authors and do not necessarily represent those of their affiliated organizations, or those of the publisher, the editors and the reviewers. Any product that may be evaluated in this article, or claim that may be made by its manufacturer, is not guaranteed or endorsed by the publisher.
